# Evaluating Trends in Mortality and Years of Life Lost Due to Cardiovascular Diseases in the Southwest of Fars Province, 2013–2023: A Cross‐Sectional Study

**DOI:** 10.1002/hsr2.71602

**Published:** 2025-12-01

**Authors:** Mostafa Eghbalian, Hossein Moameri, Neda Malek Mohammadi, Sakine Naroei, SeyedehZahra Hosseini, Mojtaba Norouzi

**Affiliations:** ^1^ Department of Public Health, School of Health; Social Determinants of Health Research Center Gonabad University of Medical Sciences Gonabad Iran; ^2^ Department of Epidemiology, School of Public Health Shahroud University of Medical Sciences Shahroud Iran; ^3^ Department of Biostatistics and Epidemiology, School of Public Health Kerman University of Medical Sciences Kerman Iran; ^4^ Psychology and Counseling Organization Tehran Iran; ^5^ Health & Medical Network Shiraz University of Medical Sciences Kazerun Iran; ^6^ Department of Epidemiology and Biostatistics, School of Health Shahrekord University of Medical Sciences Shahrekord Iran

**Keywords:** cardiovascular diseases, disease burden, premature mortality, trend, years of life lost

## Abstract

**Background and Objective:**

Cardiovascular diseases (CVDs) are the top cause of death and disability globally. Estimating the years of life lost (YLL) can inform policy and intervention programs. The present study was designed and implemented to estimate the number of deaths and YLL, estimate their trend, and examine demographic characteristics due to cardiovascular diseases.

**Material and Methods:**

This retrospective population‐based study analyzed mortality trends and YLL associated with 4314 deaths caused by CVDs. Data recorded in the Kazerun Forensic Medicine Organization were used to calculate the YLL due to cardiovascular diseases. For the descriptive analysis, we utilized the *χ* ^2^ test, considering a *p* value of less than 0.05 as statistically significant.

**Results:**

Men died from CVDs at an average age of 63.4 (±16 years), while women died at an average age of 61.5 (±17 years). CVDs caused over half (52.3%) of mortality in both sexes. Mortality from heart disease was significantly higher among individuals with lower education levels and self‐employed individuals throughout all years. The total number of YLLs due to CVDs was 43,107, which equals 14 years lost per 1000 individuals. While more men died from CVDs throughout most of the study, the difference between the number of male and female mortality narrowed in 2018 and from 2021 to 2023. According to the generalized additive model (GAM), years of life lost from heart disease mortality were increasing until almost 2019, but after 2019, this trend has been decreasing.

**Conclusion:**

The calculation of total YLL reveals a significant social burden of premature mortality from CVDs in the southwest of Fars province. This issue should be prioritized as a public health concern, necessitating preventive measures such as education, evaluation, and reduction of modifiable risk factors, increased physical activity, and blood pressure management to decrease mortality rates.

## 1 Introduction

Cardiovascular diseases (CVDs), mainly ischemic heart disease (IHD) and cerebrovascular disease, remain the leading cause of premature mortality, accounting for approximately one‐third of all deaths worldwide [1]. Despite significant advances in prevention, early diagnosis, and treatment, CVDs continue to impose a large socioeconomic burden on health systems and society, leading to reduced economic growth and deepening poverty and inequalities [2, 3, 4]. Despite a decline in standardized cardiovascular disease mortality, total global deaths from it have increased over the past two decades, more than twice as much as the total deaths from HIV, tuberculosis, and malaria [5]. Premature mortality, defined as death occurring before the expected life expectancy of a given population, is a key indicator of public health performance [6].

Mortality is the most objective measure of health problems, and Years of life lost (YLLs) are a major component of the burden of disease, especially in low‐ and middle‐income countries [7]. According to the Global Burden of Disease (GBD) framework, YLLs are calculated by multiplying the number of deaths at each age due to a specific cause by the standard life expectancy at that age [8]. This calculation essentially quantifies the number of years of life lost prematurely due to mortality. In 2019, an estimated 17.9 million individuals died from CVDs, accounting for 32% of all global deaths, and this number is expected to increase to 23.3 million by 2030 [5, 9]. Approximately 85% of these deaths are due to heart attack and stroke, and more than three‐quarters of them occur in low‐ and middle‐income countries. In 2017, the global YLLs attributable to CVDs were estimated at 330 million years [10, 11]. In Iran, a middle‐income country undergoing an epidemiological transition from communicable to noncommunicable diseases, CVDs are the leading cause of death (46% of all deaths) [12]. A previous study estimated that the 10‐year CVD risk for men and women will increase by 19.9% and 32.2%, respectively, between 2000 and 2030 [13].

Although the third Sustainable Development Goal is to reduce premature mortality from noncommunicable diseases by one‐third, and it has decreased by 17% from 2000 to 2015, this rate is not satisfactory and is not sufficient to achieve the Millennium Development Goals (SDGs) [14]. Southwest Fars Province, which includes urban and rural populations with a diverse socioeconomic mix, faces specific challenges in the health system, including limited access to specialized services, a shortage of health workforce, and disparities in service delivery between urban and rural areas [15]. Despite the high burden of CVDs in this region, local data on mortality patterns and YLLs remain limited. Furthermore, regional comparisons with national trends and appropriate intervention planning have been blocked by the lack of disaggregated evidence [16]. Therefore, calculating the years of life lost due to CVDs provides valuable information for policy decisions, design, and management of intervention programs. Because the mortality rates have changed over time with an unknown function, the nonparametric generalized additive models (GAMs) are more appropriate than the parametric models to identify the trend of mortality rates and effective factors [17]. In 2020, the GAM model was used to investigate the trend of road accidents from 2009 to 2016 [18]. Also, in another study, the GAM model was used to investigate weather and emergency call data in Hong Kong [19]. So in this study, we can use this model to examine the trend in mortality rates and the resulting burden. Therefore, the present study was conducted to estimate the number of deaths and years of life lost (YLLs) attributable to cardiovascular diseases in southwest Fars province from 2013 to 2023.

## 2 Methods

### 2.1 Study Design and Setting

A retrospective population‐based mortality trend study was conducted using death registry data from 2013 to 2023. The study focused on all registered population‐based deaths attributed to cardiovascular diseases in the southwest region of Fars province, Iran, during the study period and included both urban and rural areas.

### 2.2 Data Sources

In our study, a total of 4326 death records from CVDs were initially obtained from the electronic population‐based death registration system. After the linkage and cleaning process, including verification, removal of duplicates, and consolidation across sources, 4314 records remained for analysis. Mortality data. Mortality data were obtained from the electronic population‐based death registration system, which integrates information from several sources, with forensic medicine serving as the primary source of data. Additional sources, including hospitals, cemeteries, and urban and rural health centers, are used to increase the sensitivity and completeness of the registry. To accurately link records from these different sources, the national identification number was employed as a unique identifier, allowing for precise matching and consolidation of data. The system employs a multi‐step quality assurance process: death records reported from each source are reviewed at the health center level, where they undergo verification and cleaning. Duplicate records are identified and removed by matching key identifiers such as the father's name, date and time of death, and national identification number. Subsequently, aggregated and standardized data are registered in the national death registration system. Continuous education of personnel and strong intersectoral collaboration support ongoing improvements in data reliability. Furthermore, the cause of death coding adheres to national and international guidelines aligned with WHO standards, ensuring standardization across the data set.

### 2.3 Inclusion and Exclusion Criteria

The inclusion criteria were all deaths attributed to cardiovascular diseases, as classified in the electronic population‐based death registration system, among residents of the southwest region of Fars province between 2013 and 2023, regardless of age or sex. The exclusion criteria were duplicate records, which were identified and removed by matching on the father's name, time of death, and national identification number, as well as records where cardiovascular disease could not be confirmed as the cause of death due to insufficient information.

### 2.4 Coding of Causes of Death and Approaches to Manage Misclassification

Causes of death in our study were coded using the International Classification of Diseases, 10th Revision (ICD‐10) categories, specifically aligned with national and WHO guidelines. This coding system ensures uniform classification of CVDs. Moreover, misclassification was addressed through data verification at the health center level, including cross‐checking recorded causes against clinical and forensic sources. Death reports with insufficient information to confirm CVD as the cause were excluded. Furthermore, ongoing continuous education of registry personnel and inter‐sectoral collaboration contributed to improving the validity and precision of cause of death coding.

### 2.5 Variables and Definitions

The primary outcome variables in this study were age at death, sex, year of death, mortality due to CVDs, and YLL. Age at death represents the age in years of individuals at the time they died, providing essential information for understanding mortality distribution across different age groups. Sex categorizes individuals as male or female, an important variable given the differences in mortality patterns often observed between males and females. Year of death indicates the calendar year in which the death occurred, allowing analysis of temporal trends in mortality. Mortality due to CVDs refers to the number of deaths caused by heart and blood vessel disorders, including conditions such as coronary heart disease and stroke. In this study, the term “undergraduate” refers to individuals who hold a bachelor's degree, which in Iran typically requires completion of a 4‐year academic program at a university. The standard life expectancy indicates the maximum possible lifespan that an individual can expect to live in good health, without avoidable risks or serious injuries, and with access to adequate healthcare. This serves as a benchmark for calculating YLL due to early death. YLL quantifies premature mortality by measuring the years of life lost when a death occurs before the expected standard lifespan is reached.

### 2.6 Statistical Analysis

First, using graphs and descriptive statistics, the trend in the mortality ratio was illustrated in relation to demographic variables. The subsequent formula was employed to determine the years of life lost as a result of deaths caused by cardiovascular diseases [20]:

$$\mathrm{YLL}={\mathrm{NC}}^{\left(\mathrm{ra}\right)}/{\left(\beta +r\right)}^{2}[e^{-\left(\beta +r\right)\left(L+a\right)}\left[-\left(\beta +r\right){\left(L+a\right)}_{1}\right]-e^{-\left(\beta +r\right)a}[-\left(\beta +r\right)a-1]]$$

Where *N* is the number of deaths at a certain age and gender. *L* is the standard of living of the deceased at the same age and gender. *r* is the Discounting Rate, which is equal to 0.03. *β* is the contract rate in calculating the age value, equal to 0.04. *C* is a modified constant value equal to 0.1658. *a* is the age at which death occurred, and *e* denotes Euler's number (≈2.718). The main idea behind the YLL calculation is to estimate the total number of years that are prematurely lost due to early death by considering the age at death, the expected remaining years based on life expectancy, and adjustments for factors like quality of life and standardization.

The formula begins with the number of deaths at each age, multiplies that by the expected remaining years of life for those individuals, incorporates adjustments for economic and demographic variables using standard constants, and then applies a discount rate to reflect the present value of future years lost. To ensure standardization, the standard population data from 2013 for low‐ and middle‐income countries was used [21].

The adjusted mortality ratio by gender was also presented using a graph. Finally, using the GAM model, the mortality trend over time was presented. To assess the trend and pattern of the accidents, the GAM for the mortality rate was used. The response variable in this study is the rate of mortality due to heart disease. In statistics, an additive model is a regression model in which the response variable depends linearly on the unknown smooth functions of certain predictor variables [22]. All figures and statistical analyses were performed in R.4.5.1 software, and the “gam” command was used to fit the model. For descriptive analysis, the *χ* ^2^ test was applied, with statistical significance determined at a *p* value threshold of less than 0.05.

### 2.7 Ethical Statement

The data used in this study came from a confidential source and were accessed as per data use and confidentiality agreements. The retrospective study received ethical approval from Shiraz University of Medical Sciences' Institutional Review Board (IRB) (Approval ID: IR.SUMS. REC.1399.859) before initiation of study activities.

## 3 Result

**Figure 1:**
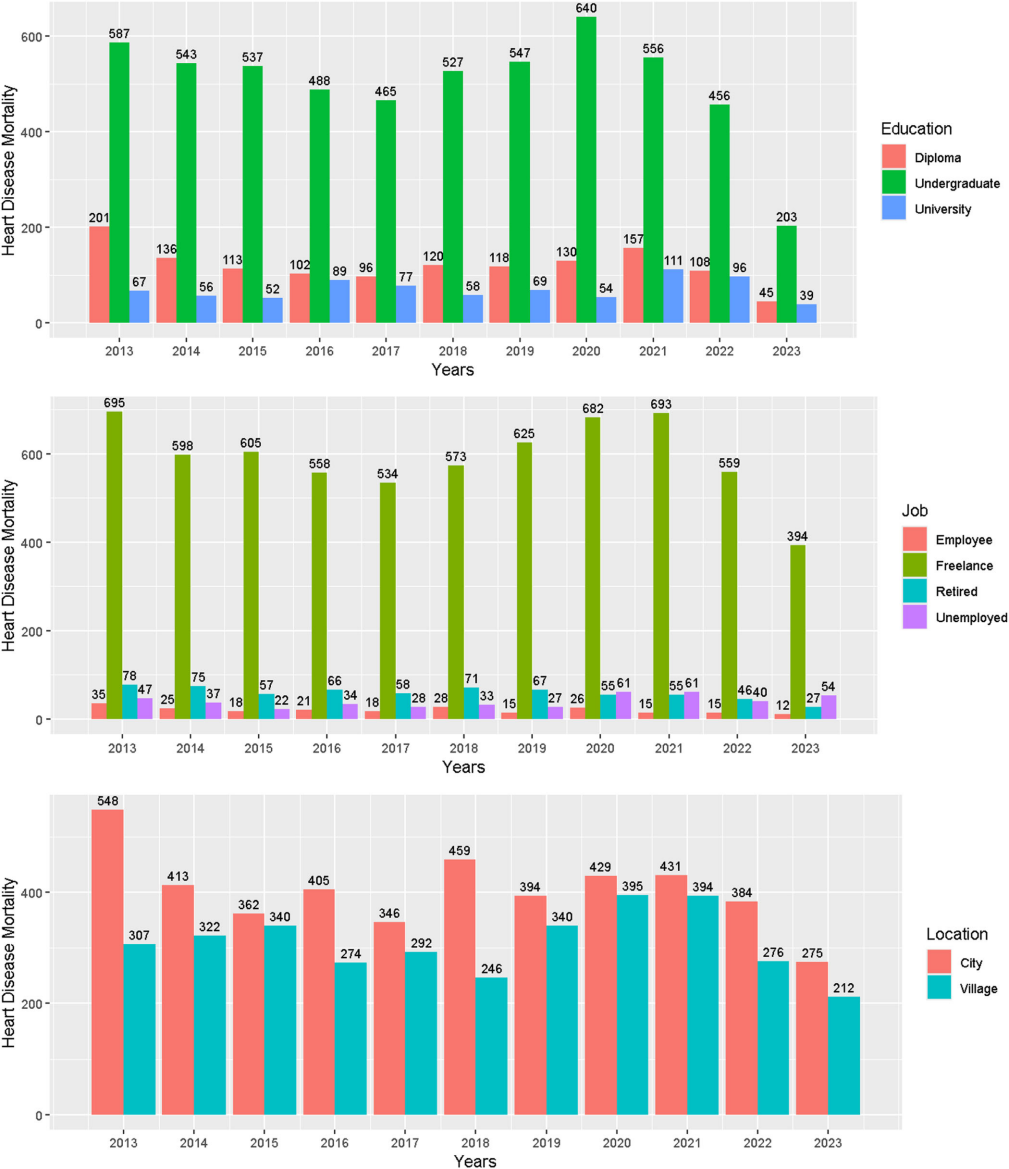
Mortality trends due to cardiovascular diseases by education, occupation, and place of residence in the southwest of Fars province, 2013–2023.

Based on the results of the years studied, 4314 deaths due to cardiovascular diseases occurred, of which 2303 were in men and 2011 in women. The average age of mortality from cardiovascular diseases was 63.35 ± 15.96 for men and 61.53 ± 16.61 for women, respectively. According to Figure 1, the number of deaths from heart disease among undergraduate patients was significantly higher than among educated patients throughout all years. Also, throughout all years, university graduates had lower mortality rates than diploma holders. Also, the mortality trend over the years differed in education status (*X*‐squared = 5374.3, *p* value < 2.2e−16). Self‐employed patients, followed by retirees (except in 2020, 2021, and 2023), had the highest mortality rates across all years (X‐squared = 14,103, *p* value < 2.2e‐16). Urban dwellers also had higher mortality rates throughout all years. Also, patients living in cities had higher mortality rates than patients living in rural areas throughout all years (X‐squared = 267.52, *p* value < 2.2e‐16).

**Figure 2:**
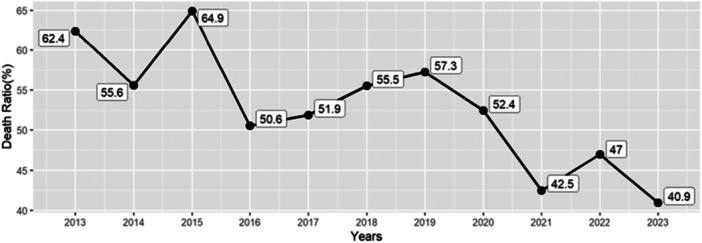
Trend in proportional mortality rate due to cardiovascular diseases in the southwest of Fars province, 2013–2023.

Between 2013 and 2023, the mortality rate from cardiovascular diseases in the southwest region of Fars province experienced volatility, starting at 62% in 2013, dropping to about 51% in 2016 and 2017, then increasing to 57% in 2019, and finally dropping steeply to 41% in 2023. The overall decline in the final years of the observation period suggests improvements in interventions targeted at the cardiovascular system, or perhaps the causes of mortality had shifted during the period (Figure 2).

During the COVID‐19 pandemic, particularly between 2019 and 2021, we saw the highest crude and age‐standardized mortality rates recorded. In the given time frame, total YLL was 43,107 (14 per 1000 individuals) in both sexes (Table 1). This was an effect of the indirect and direct factors described. Directly, there was an increase in mortality due to infections of SARS‐CoV‐2. Indirectly, there was an increase in mortality due to the neglect of treatment for cardiovascular diseases and the pandemic‐induced deterioration of fundamental health conditions. The increase in mortality and YLL can, therefore, be attributed to direct effects of infections and the indirect impact of the pandemic on healthcare systems. Additional details can be found in the Supporting Information S1.

**Table 1:** Crude and standardized mortality rate (per 100,000 population) and YLL due to cardiovascular diseases by sex and year in the southwest of Fars province, 2013–2023.

Year	No. death	Crude mortality rate Per (10,000)	Age‐standardized rate (95%CI) per (100,000)	YLL	
				No.	Per (1000)
	Both sex	Both sex	Both sex	Both sex	Both sex
2013	855	28	38.3 (35.8–40.9)	4935	16
2014	735	24	32.8 (30.4–35.1)	5595	18
2015	702	23	32.5 (30.1–34.9)	4312	14
2016	679	22	35.2 (32.6–37.9)	3775	14
2017	638	21	34.8 (32.1–37.5)	3122	12
2018	705	23	38.2 (35.4–41.0)	3657	14
2019	734	24	40.5 (37.5–43.4)	3528	13
2020	824	27	45.4 (42.3–48.5)	4236	16
2021	825	27	45.4 (42.3–48.5)	4127	16
2022	660	21	36.4 (33.6–39.1)	3395	13
2023	487	16	35.1 (32.0–38.2)	2425	12
Total	7844	26	37.5 (36.7–38.4)	43,107	14

**Figure 3:**
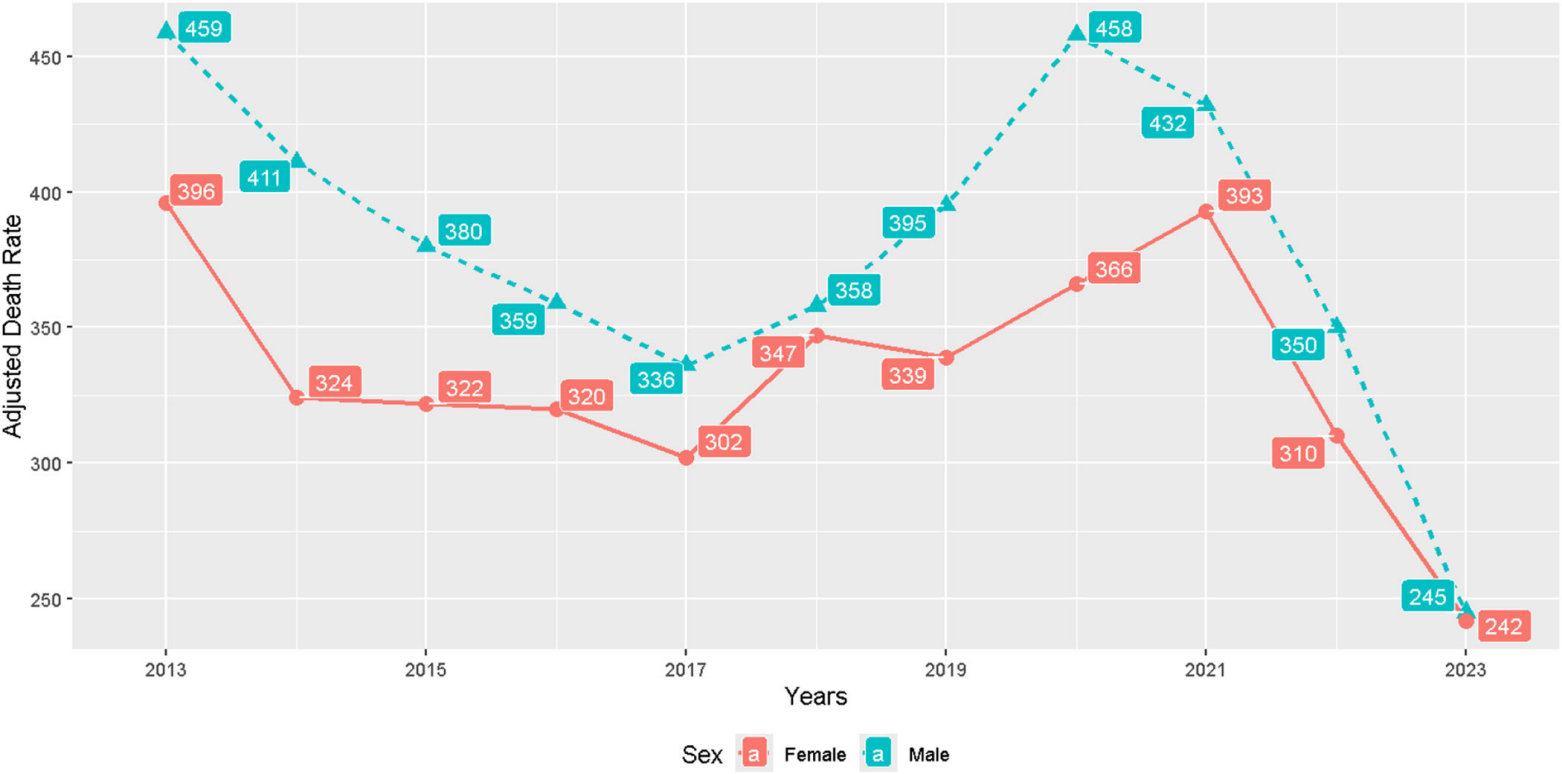
Trends of adjusted mortality ratio because of cardiovascular diseases by sex in the southwest of Fars province, 2013–2023.

Figure 3 illustrates the trends in adjusted mortality rates due to cardiovascular diseases in the southwest of Fars province from 2013 to 2023, with males consistently showing higher mortality rates than females throughout the period. However, from 2018 onwards, this sex difference in mortality rates began to diminish, with rates converging closely by 2023, indicating a narrowing gap in cardiovascular mortality between men and women. Both sexes exhibited fluctuation in mortality rates over the years, with notable peaks around 2013 and during 2019–2021, before a decline towards 2023. This trend reflects changing patterns in cardiovascular health outcomes in this region over the past decade.

**Figure 4:**
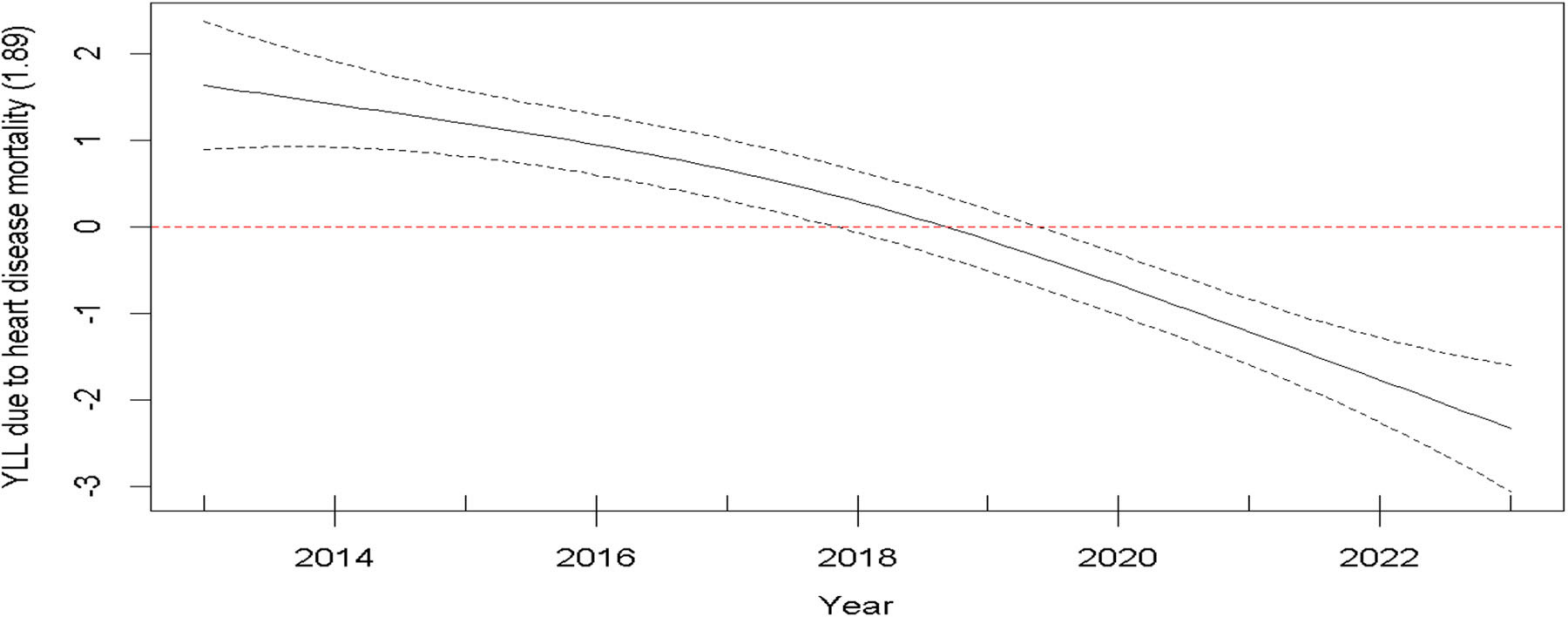
Examining the trends in years of life lost due to heart disease mortality in the southwest of Fars province, 2013–2023.

The picture shows the changes in YLL from heart disease deaths in the southwest part of Fars province over the years 2013–2023. When the line is below zero, it means a downward change; when it's above zero, it means an upward change. If the line gets closer to zero, that says that the rate is changing less fast, but if it moves further away from zero, that tells us the rate is changing more quickly. It shows YLL was going up but slowing down until about 2019, and then after that point began to go down. This means fewer early deaths from heart disease were happening at a quicker pace till 2019, before they started falling (Figure 4).

## 4 Discussion

Overall, the present study found that CVDs accounted for 52.3% of all‐cause mortality in both sexes. Furthermore, the mortality rate and YLL because of CVDs were continuously greater in men than in women throughout the study period. Despite fluctuations during the years of study, the mortality rate from CVDs showed a general decrease. Specifically, the CVD mortality rate was 62% in 2013 but decreased to 57% in 2019 and 41% in 2023. In addition, the total YLL due to CVDs was estimated to be 43,107 (14 per 1000 individuals) for both sexes. Analysis of the YLL trend indicates a rising rate of YLL caused by CVDS mortality until 2019, followed by a decreasing trend thereafter.

The findings of this study demonstrate that CVDs were the major cause of mortality in both sexes, accounting for 52.3% of all deaths. This finding is consistent with a previous study in the same geographic area. For example, studies in Fars Province have highlighted the significant impact of cardiovascular mortality, reporting large numbers of deaths due to ischemic heart disease over many years [23] and identifying CVD as the main cause of mortality to YLL, in addition to other major noncommunicable diseases in the region [24]. Furthermore, Mirahmadizadeh et al. demonstrated that CVDs contribute to the highest death rate and the largest number of YLL [16]. The significant prevalence is scientifically supported by the region's demographic and epidemiological transition, reflecting national trends where non‐communicable diseases have overtaken communicable diseases as the leading cause of death. This shift is often linked to increased life expectancy and higher rates of established risk factors, such as hypertension and dyslipidemia, especially in urban centers like Shiraz [16, 25]. Targeted, evidence‐based interventions tailored to local epidemiology are crucial. This demands a two‐pronged approach [1]: promoting urban planning that encourages active transportation and accessible green spaces to address lifestyle‐related risk factors, and [2] strengthening the primary healthcare system by providing immediate, standardized training for family physicians in aggressive CVD risk factor management, especially hypertension and dyslipidemia. Additionally, investing in data‐driven public awareness campaigns that are adapted to local demographics and gender‐specific needs is vital.

The mortality rate and YLL due to CVDs were higher in men than women throughout the study period, which is consistent with the findings of the study by Sharifi et al. and the Global Burden of Disease study of CVDs in the Eastern Mediterranean region [23, 26, 27]. The gender gap in mortality from CVDs and YLL can be attributed to factors such as obesity, high blood pressure, high cholesterol, alcohol consumption, and tobacco use [28]. These variables are more prevalent in men [29]. Additionally, local data from Shiraz province, including the Shiraz Heart Study and a cohort of Shiraz University employees, showed that men have significantly higher Atherosclerotic Cardiovascular Disease (ASCVD) risk scores than women (38.5% vs. 6.3%), with a significant proportion of participants having uncontrolled hypertension and diabetes, emphasizing the importance of targeted prevention strategies in the local population [30, 31]. Therefore, strategies to address CVDs among men in Fars Province include targeted screening programs, gender‐sensitive health education and behavioral interventions, stronger alcohol and tobacco control laws, improved access to high‐quality healthcare, increased research into the sources of the problem, and enhanced collaboration between governmental and nongovernmental organizations to implement prevention initiatives.

The age‐standardized mortality rate has declined while the total number of crude CVD deaths has increased. Azarbakhsh's study showed similar results to ours. They observed a decreasing trend in standardized mortality rates in Fars Province [7]. This apparent paradox is not a conflict; rather, it clearly reflects a significant demographic shift toward an older population in the region, which is common in areas experiencing a successful but incomplete epidemiological transition. The decline in ASR indicates that intervention measures, such as improved medical care for diseases like AMI [32, 33] and localized early diagnosis, are effectively reducing age‐specific mortality risks. However, the rising crude count shows that the benefits of risk reduction are being offset by an increasing number of older individuals, who naturally have a higher baseline mortality risk, regardless of improved survival within their age group. Therefore, the hypothesis of an overall reduction in CVD mortality in Iran is not entirely valid. Although some conditions, such as acute myocardial infarction, decreased from 2006 to 2017, this trend cannot be applied to all types of CVDs [32, 33]. Furthermore, a study in Fars Province showed fluctuations in the standardized mortality rate over 16 years (2004–2019) [34]. However, the increased incidence of major risk factors such as hypertension, diabetes, and obesity, which are mostly caused by lifestyle and nutritional changes, remains a serious concern [35]. While improvements in medical care, access to healthcare services, and public awareness have contributed to lower age‐specific risks, regional disparities in case fatality and healthcare accessibility continue to influence overall CVD mortality. Despite these challenges, ongoing efforts in prevention, health promotion, and healthcare delivery are helping to enhance cardiovascular health in Fars Province.

In our study, cardiovascular diseases accounted for 43,107 YLL, which is considerably higher than the findings from Northeastern Iran (1438 YLL due to CVDs), but lower than the figures reported in Japan [36, 37]. indicate geographical variations in cardiovascular mortality rates. However, the key factor is that the high YLL in Fars Province might be caused by local factors such as rapid urbanization, increasing obesity, hypertension, and diabetes rates, along with changes in dietary habits and physical activity patterns [38]. Additionally, socioeconomic disparities, including variations in income and education, affect awareness and timely access to prevention and treatment options. Furthermore, restricted access to specialized cardiovascular care in Fars' rural areas may result in increased YLL [39]. Therefore, although international and national comparisons are valuable, understanding these findings within the local context emphasizes the urgent need for targeted prevention and better healthcare coverage to reduce premature cardiovascular deaths in this region.

Our analysis indicated that years lost due to CVDs are decreasing, which is consistent with recent investigations in Iranian populations. For instance, a national study showed that men and diabetic individuals experienced a decrease in CVD risk [40]. However, these improvements might not lead to long‐term relief, as other studies predict that demographic shifts, especially an aging population, will significantly increase the CVD burden in Iran. A modeling study forecasted that years of life lost to CVDs could more than double, increasing from 847,309 disability‐adjusted life years (DALYs) in 2005 to 1,728,836 DALYs by 2025 [41]. In Fars Province, this warning is especially important due to the region's rapidly aging population, uneven distribution of healthcare resources, and high rate of modifiable risk factors. Although current trends show some progress, the burden of CVDs in Fars is likely to rise significantly in the coming years without stronger preventive efforts and targeted interventions suitable to the local context. Policymakers need to prioritize filling the gaps in the basic healthcare infrastructure and the basic healthcare resource allocation in the identified underserved locations. Relevant targeted risk preventative initiatives should include the culturally suitable promotion and the targeting of positive dietary and physical activity interventions aimed at hypertension, diabetes, and obesity. Given the future burden of CVD, the public health education concerning lifestyle modifications pegged to the risks of CVD should go hand in hand with the increased efforts around screening, early detection, and intervention services.

There are various limitations to this study regarding the calculation and the data used for YLL. First, the estimates of YLL calculations do not incorporate the changes in the incidence and mortality rates, which can be biased as a result of changes in various risk factors, health care, and interventions that might occur within the time period of a study. Furthermore, the growth and changes in the structure of the population, such as aging, growth, and demographic shifts, can affect the number and distribution of deaths, which have not been considered in the estimates. Therefore, the generalizability of the YLLs could be impacted negatively. In addition, access to more detailed information on deaths YLL within the various subtypes of cardiovascular diseases in the electronic population‐based death registration system meant that the analysis had to be confined to the broader category of cardiovascular diseases. Finally, the lack of examination of potential underreporting and misclassification of the death registries might have biased the study. The estimate of disease burden also might not reflect the true disparities, as the analysis has been based on only the most basic education and occupational data. Incorporating better clinical data, registry data, and socioeconomic data in future studies would improve the assessment of cardiovascular mortality and provide greater understanding and more detailed insights.

## 5 Conclusion

The current study found that CVDs are the main causes of mortality among both genders, with a particularly high impact on men. Although mortality rates have fluctuated over the years, there has been an overall decline. The analysis of YLL trends showed an increase in YLL associated with CVD mortality until 2019, followed by a subsequent decline. This indicates a need for targeted interventions, including preventive interventions, improved access to healthcare, and comprehensive health education to raise awareness and encourage behavioral changes related to cardiovascular health.

## Author Contributions

All authors contributed to the study's conception and design. Material preparation, data collection, and analysis were performed by M.E, M.N, and S.H. All authors read and approved the final manuscript. Writing – review and editing by M.E, M.N, H.M, N.M, and S.N.

## Conflicts of Interest

The authors declare no conflicts of interest.

## Transparency Statement

The corresponding author, Mojtaba Norouzi, affirms that this manuscript is an honest, accurate, and transparent account of the study being reported; that no important aspects of the study have been omitted; and that any discrepancies from the study as planned (and, if relevant, registered) have been explained.

## Supporting information

supplementary.

## Data Availability

The data that support the findings of this study are available from the corresponding author upon reasonable request.
