# Supplementary material for: Evaluating Trends in Mortality and Years of Life Lost Due to Cardiovascular Diseases in the Southwest of Fars Province, 2013–2023: A Cross‐Sectional Study
Source: Health Sci Rep. 2025 Dec 1;8(12):e71602. doi: 10.1002/hsr2.71602 (PMC12668901; doi:10.1002/hsr2.71602)
Supplement: Supplementary file 1 — supplementary. [file HSR2-8-e71602-s001.pdf]

Year	No. death			Crude mortality rate Per (10000)			ASR (95%CI) Per (100000)			YLL			YLL Per (1000)		
	Male	Female	Both	Male	Female	Both	Male	Female	Both	Male	Female	Both	Male	Female	Both
2013	459	396	855	28	57	28	39.5(35.9-43.1)	37.1(33.4-40.7)	38.3(35.8-40.9)	2767	2168	4935	17	15	16
2014	411	324	735	25	49	24	35.2(31.8-38.6)	30.1(26.9-33.4)	32.8(30.4-35.1)	3210	2385	5595	20	16	18
2015	380	322	702	24	47	23	34.0(30.6-37.5)	30.9(27.5-34.3)	32.5(30.1-34.9)	2463	1849	4312	16	12	14
2016	359	320	679	22	46	22	36.4(32.7-40.2)	33.9(30.2-37.7)	35.2(32.6-37.9)	2269	1506	3775	16	11	14
2017	336	302	638	21	43	21	35.9(32.1-39.7)	33.6(29.8-37.4)	34.8(32.1-37.5)	1706	1416	3122	13	11	12
2018	358	347	705	22	47	23	38.0(34.1-41.9)	38.4(34.3-42.4)	38.2(35.4-41.0)	2077	1580	3657	15	12	14
2019	395	339	734	24	49	24	42.5(38.4-46.7)	38.3(34.2-42.3)	40.5(37.5-43.4)	2023	1505	3528	15	12	13
2020	458	366	824	28	55	27	49.3(44.8-53.9)	41.3(37.1-45.5)	45.4(42.3-48.5)	2673	1563	4236	20	12	16
2021	432	393	825	27	55	27	46.4(42.0-50.8)	44.3(40.0-48.7)	45.4(42.3-48.5)	2408	1719	4127	18	13	16
2022	350	310	660	22	44	21	37.7(33.8-41.7)	35.0(31.1-38.9)	36.4(33.6-39.1)	2090	1305	3395	16	10	13
2023	245	242	487	15	33	16	34.5(30.2-38.8)	35.7(31.2-40.2)	35.1(32.0-38.2)	1369	1056	2425	13	10	12
Total	4183	3661	7844	27	25	26	39.0(37.8-40.1)	36.1(34.9-37.2)	37.5(36.7-38.4)	25055	18052	43107	16	12	14
